# 6-[4-(Diphenyl­amino)­phen­yl]quinoline 1-oxide

**DOI:** 10.1107/S1600536812031662

**Published:** 2012-07-25

**Authors:** Li-Zhi Wang, Yun Chi, Xiang-Xiang Li, Jian-Ning Guan

**Affiliations:** a College of Science, Nanjing University of Technology, Xinmofan Road No. 5 Nanjing, Nanjing 210009, People’s Republic of China

## Abstract

In the title mol­ecule, C_27_H_20_N_2_O, a triphenyl­amine derivative of quinoline, the three benzene rings linked through an N atom form a propeller shape, with dihedral angles between the mean planes of pairs of rings of 75.57 (9), 55.68 (9) and 83.66 (9)°. The quinoline ring is essentially planar, with an r.m.s. deviation of the fitted atoms of 0.0155 Å, and forms a dihedral angle of 33.52 (8)° with the benzene ring to which it is bonded. Weak C—H⋯π inter­actions are also observed in the crystal structure.

## Related literature

For background to triphenyl­amine derivatives, see: Lin *et al.* (2010 ▶). For preparation, see: Liu *et al.* (2011 ▶). For the crystal structure of a related compound, see: Xie *et al.* (2011 ▶).
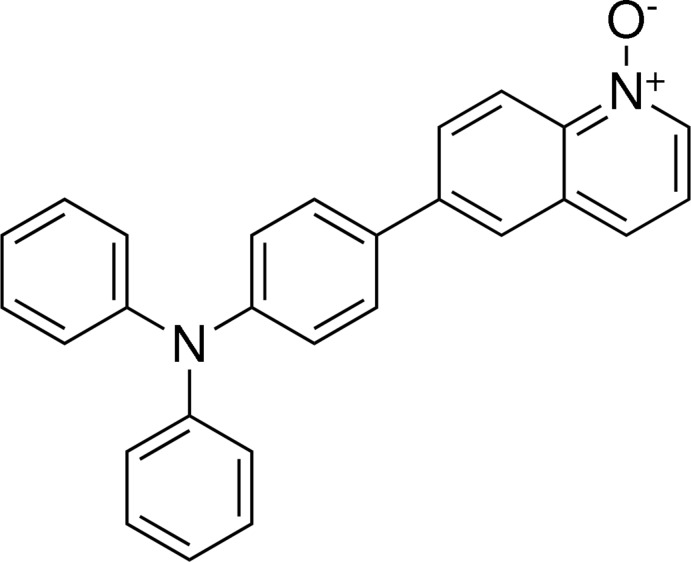


## Experimental

### 

#### Crystal data

C_27_H_20_N_2_O
*M* *_r_* = 388.45
Monoclinic, 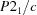

*a* = 16.774 (3) Å
*b* = 9.6130 (19) Å
*c* = 13.253 (3) Å
β = 107.05 (3)°
*V* = 2043.1 (7) Å^3^
*Z* = 4
Mo *K*α radiation
μ = 0.08 mm^−1^
*T* = 293 K
0.30 × 0.20 × 0.10 mm

#### Data collection

Enraf–Nonius CAD-4 diffractometer
Absorption correction: ψ scan (North *et al.*, 1968 ▶) *T*_min_ = 0.977, *T*_max_ = 0.992
3881 measured reflections
3747 independent reflections
2263 reflections with *I* > 2σ(*I*)
*R*_int_ = 0.072
3 standard reflections every 200 reflections intensity decay: 1%

#### Refinement

*R*[*F* ^2^ > 2σ(*F* ^2^)] = 0.058
*wR*(*F* ^2^) = 0.156
*S* = 1.00
3747 reflections
271 parameters
H-atom parameters constrained
Δρ_max_ = 0.20 e Å^−3^
Δρ_min_ = −0.16 e Å^−3^

### 

Data collection: *CAD-4 EXPRESS* (Enraf–Nonius, 1994 ▶); cell refinement: *CAD-4 EXPRESS*; data reduction: *XCAD4* (Harms & Wocadlo, 1995 ▶); program(s) used to solve structure: *SHELXS97* (Sheldrick, 2008 ▶); program(s) used to refine structure: *SHELXL97* (Sheldrick, 2008 ▶); molecular graphics: *SHELXTL* (Sheldrick, 2008 ▶); software used to prepare material for publication: *PLATON* (Spek, 2009 ▶).

## Supplementary Material

Crystal structure: contains datablock(s) global, I. DOI: 10.1107/S1600536812031662/pv2553sup1.cif

Structure factors: contains datablock(s) I. DOI: 10.1107/S1600536812031662/pv2553Isup2.hkl

Supplementary material file. DOI: 10.1107/S1600536812031662/pv2553Isup3.cml

Additional supplementary materials:  crystallographic information; 3D view; checkCIF report

## Figures and Tables

**Table 1:** Hydrogen-bond geometry (Å, °) *Cg*3 is the centroid of the C7–C12 benzene ring.

*D*—H⋯*A*	*D*—H	H⋯*A*	*D*⋯*A*	*D*—H⋯*A*
C17—H17*A*⋯*Cg*3^i^	0.93	2.76	3.640 (3)	158

Symmetry code: (i) 
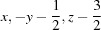
.

**Fig. 1.**
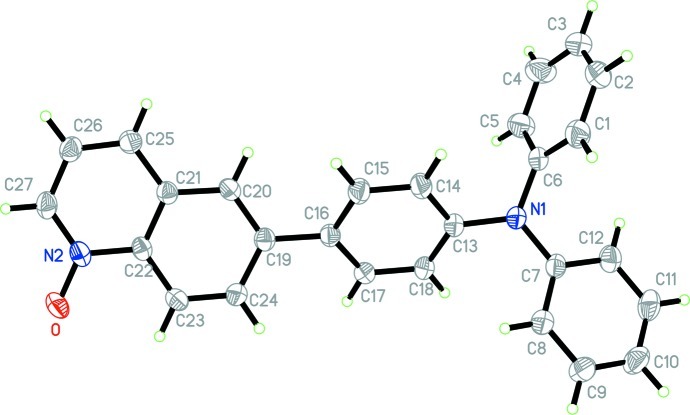
The molecular structure of the title molecule showing the atom-numbering scheme and displacement ellipsoids at the 30% probability level.

**Fig. 2.**
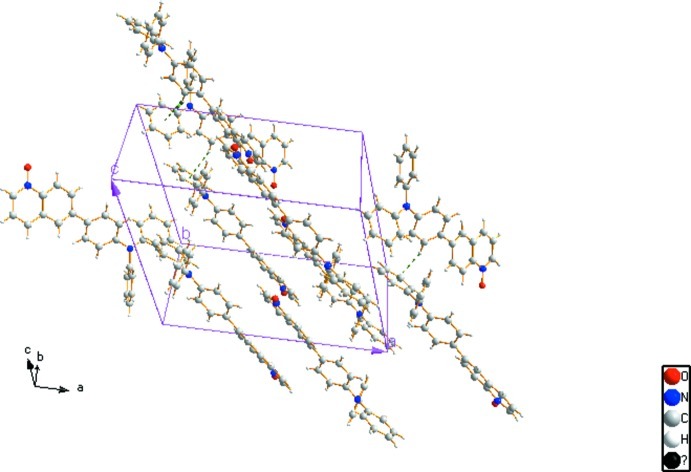
A packing diagram of the title compound. The weak C—H···π interactions are shown as dashed lines.

## supplementary crystallographic information

### Comment

Triphenylamine (TPA) derivatives, are important structural motifs in numerous dye-sensitized solar cells (Lin *et al.*, 2010). As a part of our studies on the synthesis of TPA derivatives, the title compound was synthesized (Liu *et al.*, 2011). We report herein the crystal structure of the title compound.

In the title molecule (Fig. 1), the quinoline ring (N2/C19–C27) is essentially planar with rmsd of the fitted atoms 0.0155 Å. The benzene rings bonded to the central N1 atom form a propeller with dihedral angles between the mean planes being 75.57 (9), 55.68 (9) and 83.66 (9)° between the pairs of rings: C1–C6/C7–C12, C7–C12/C13–C18 and C1–C6/C13–C18, respectively. The dihedral angle between the mean planes of quinoloine and benzene ring (C13–C18) is 33.52 (8)°. The bond lengths and bond angles in the title compound agree with the corresponding bond lengths and angles reported for a closely related compound (Xie *et al.*, 2011).

The crystal structure is consolidated by weak C—H···π interactions C7—H17A···Cg3; Cg3 is the centroid of the C7–C12 ring (Fig. 2 and Table 1).

### Experimental

A mixture of 6-bromoquinoline (0.25 mmol), [4-(diphenylamino)phenyl]boronic acid (0.375 mmol), Pd(OAc)_2_ (0.5 mol-%), K_3_PO_4_.7H_2_O (0.5 mmol), distilled water (0.65 ml) and *i*PrOH (1.35 ml) was stirred at room temperature in air for 1 h. The mixture was added to brine (15 ml) and extracted four times with ethyl acetate (415 ml). The solvent was evaporated under vacuum, and then subjected to short-column chromatography on silica gel (200–300 mesh) to get N,N-diphenyl-4-(quinolin-6-yl)aniline (yield = 88.4%, m.p. 413–414 K). N,N-diphenyl-4-(quinolin-6-yl)aniline (6.55 g, 22.3 mmol) was reacted with 3-chloroperoxybenzoic acid (*m*CPBA) (4.92 g, 24.5 mmol) in dichloromethane (80 ml). After 12 h stirring at room temperature, the reaction mixture was concentrated and the solid thus obtained was filtered and recrystallized from ethyl acetate to get the title compound (yield = 92.5%, m.p. 461–463 K).

### Refinement

All H atoms were placed geometrically with C—H = 0.93 Å and refined as riding with *U*_iso_(H) = 1.2*U*_eq_(C).

### Crystal data

**Table:**

C_27_H_20_N_2_O	*F*(000) = 816
*M**_r_* = 388.45	*D*_x_ = 1.263 Mg m^−^^3^
Monoclinic, *P*2_1_/*c*	Mo *K*α radiation, λ = 0.71073 Å
Hall symbol: -P 2ybc	Cell parameters from 25 reflections
*a* = 16.774 (3) Å	θ = 9–13°
*b* = 9.6130 (19) Å	µ = 0.08 mm^−^^1^
*c* = 13.253 (3) Å	*T* = 293 K
β = 107.05 (3)°	Block, colourless
*V* = 2043.1 (7) Å^3^	0.30 × 0.20 × 0.10 mm
*Z* = 4	

### Data collection

**Table:**

Enraf–Nonius CAD-4 diffractometer	2263 reflections with *I* > 2σ(*I*)
Radiation source: fine-focus sealed tube	*R*_int_ = 0.072
Graphite monochromator	θ_max_ = 25.4°, θ_min_ = 1.3°
ω/2θ scans	*h* = −20→0
Absorption correction: ψ scan (North *et al.*, 1968)	*k* = 0→11
*T*_min_ = 0.977, *T*_max_ = 0.992	*l* = −15→15
3881 measured reflections	3 standard reflections every 200 reflections
3747 independent reflections	intensity decay: 1%

### Refinement

**Table:**

Refinement on *F*^2^	Primary atom site location: structure-invariant direct methods
Least-squares matrix: full	Secondary atom site location: difference Fourier map
*R*[*F*^2^ > 2σ(*F*^2^)] = 0.058	Hydrogen site location: inferred from neighbouring sites
*wR*(*F*^2^) = 0.156	H-atom parameters constrained
*S* = 1.00	*w* = 1/[σ^2^(*F*_o_^2^) + (0.078*P*)^2^] where *P* = (*F*_o_^2^ + 2*F*_c_^2^)/3
3747 reflections	(Δ/σ)_max_ < 0.001
271 parameters	Δρ_max_ = 0.20 e Å^−^^3^
0 restraints	Δρ_min_ = −0.16 e Å^−^^3^

### Special details

**Table:**

Geometry. All esds (except the esd in the dihedral angle between two l.s. planes) are estimated using the full covariance matrix. The cell esds are taken into account individually in the estimation of esds in distances, angles and torsion angles; correlations between esds in cell parameters are only used when they are defined by crystal symmetry. An approximate (isotropic) treatment of cell esds is used for estimating esds involving l.s. planes.
Refinement. Refinement of F^2^ against ALL reflections. The weighted R-factor wR and goodness of fit S are based on F^2^, conventional R-factors R are based on F, with F set to zero for negative F^2^. The threshold expression of F^2^ > 2sigma(F^2^) is used only for calculating R-factors(gt) etc. and is not relevant to the choice of reflections for refinement. R-factors based on F^2^ are statistically about twice as large as those based on F, and R-factors based on ALL data will be even larger.

### Fractional atomic coordinates and isotropic or equivalent isotropic displacement parameters (Å^2^)

**Table:**

	*x*	*y*	*z*	*U*_iso_*/*U*_eq_	
O	0.46148 (12)	−0.0633 (2)	−0.26382 (13)	0.0624 (5)	
N1	0.17381 (14)	0.1024 (2)	0.34131 (16)	0.0563 (6)	
C1	0.22097 (18)	0.0401 (3)	0.5285 (2)	0.0637 (8)	
H1A	0.2412	0.1298	0.5452	0.076*	
N2	0.47292 (13)	−0.1387 (2)	−0.17945 (15)	0.0471 (5)	
C2	0.2295 (2)	−0.0578 (4)	0.6077 (2)	0.0727 (9)	
H2A	0.2556	−0.0333	0.6775	0.087*	
C3	0.1999 (2)	−0.1898 (3)	0.5843 (3)	0.0747 (9)	
H3A	0.2055	−0.2551	0.6378	0.090*	
C4	0.1622 (2)	−0.2248 (3)	0.4821 (3)	0.0796 (9)	
H4A	0.1420	−0.3146	0.4657	0.096*	
C5	0.1536 (2)	−0.1290 (3)	0.4029 (2)	0.0670 (8)	
H5A	0.1279	−0.1549	0.3333	0.080*	
C6	0.18249 (16)	0.0043 (3)	0.42509 (19)	0.0497 (7)	
C7	0.12495 (15)	0.2234 (3)	0.33659 (18)	0.0471 (6)	
C8	0.13847 (16)	0.3420 (3)	0.2849 (2)	0.0538 (7)	
H8A	0.1800	0.3422	0.2514	0.065*	
C9	0.09116 (19)	0.4601 (3)	0.2823 (2)	0.0653 (8)	
H9A	0.1002	0.5384	0.2459	0.078*	
C10	0.0310 (2)	0.4622 (3)	0.3332 (2)	0.0721 (9)	
H10A	−0.0001	0.5426	0.3325	0.086*	
C11	0.01655 (17)	0.3467 (3)	0.3850 (2)	0.0667 (8)	
H11A	−0.0246	0.3487	0.4191	0.080*	
C12	0.06248 (16)	0.2266 (3)	0.3871 (2)	0.0572 (7)	
H12A	0.0519	0.1481	0.4221	0.069*	
C13	0.21718 (15)	0.0743 (3)	0.26611 (19)	0.0475 (6)	
C14	0.29592 (17)	0.0150 (3)	0.29892 (19)	0.0558 (7)	
H14A	0.3203	−0.0034	0.3702	0.067*	
C15	0.33867 (16)	−0.0169 (3)	0.22768 (19)	0.0539 (7)	
H15A	0.3917	−0.0554	0.2519	0.065*	
C16	0.30402 (15)	0.0074 (3)	0.12001 (18)	0.0429 (6)	
C17	0.22541 (15)	0.0677 (2)	0.08783 (19)	0.0452 (6)	
H17A	0.2012	0.0869	0.0166	0.054*	
C18	0.18226 (15)	0.0998 (3)	0.15911 (19)	0.0485 (6)	
H18A	0.1293	0.1389	0.1351	0.058*	
C19	0.34862 (14)	−0.0321 (2)	0.04275 (18)	0.0426 (6)	
C20	0.39816 (15)	−0.1491 (2)	0.05721 (18)	0.0454 (6)	
H20A	0.4031	−0.2046	0.1162	0.054*	
C21	0.44174 (14)	−0.1874 (2)	−0.01543 (18)	0.0420 (6)	
C22	0.43129 (14)	−0.1041 (2)	−0.10499 (17)	0.0411 (6)	
C23	0.38061 (15)	0.0147 (3)	−0.12111 (18)	0.0463 (6)	
H23A	0.3742	0.0696	−0.1807	0.056*	
C24	0.34094 (15)	0.0486 (3)	−0.04859 (18)	0.0464 (6)	
H24A	0.3076	0.1277	−0.0595	0.056*	
C25	0.49522 (16)	−0.3042 (2)	0.0002 (2)	0.0498 (6)	
H25A	0.5029	−0.3606	0.0593	0.060*	
C26	0.53503 (16)	−0.3321 (3)	−0.0729 (2)	0.0521 (7)	
H26A	0.5707	−0.4082	−0.0634	0.062*	
C27	0.52346 (16)	−0.2490 (3)	−0.1614 (2)	0.0522 (7)	
H27A	0.5519	−0.2708	−0.2100	0.063*	

### Atomic displacement parameters (Å^2^)

**Table:**

	*U*^11^	*U*^22^	*U*^33^	*U*^12^	*U*^13^	*U*^23^
O	0.0795 (13)	0.0698 (13)	0.0483 (11)	0.0007 (11)	0.0350 (10)	0.0082 (9)
N1	0.0731 (15)	0.0497 (13)	0.0605 (14)	0.0173 (11)	0.0418 (12)	0.0101 (11)
C1	0.076 (2)	0.0584 (18)	0.0597 (18)	−0.0005 (15)	0.0241 (16)	−0.0076 (14)
N2	0.0530 (13)	0.0484 (13)	0.0458 (12)	−0.0104 (11)	0.0237 (10)	−0.0059 (10)
C2	0.089 (2)	0.080 (2)	0.0463 (16)	0.0161 (19)	0.0160 (16)	0.0009 (16)
C3	0.094 (2)	0.068 (2)	0.070 (2)	0.0229 (19)	0.0360 (19)	0.0199 (17)
C4	0.109 (3)	0.0521 (18)	0.076 (2)	−0.0056 (18)	0.024 (2)	0.0076 (16)
C5	0.088 (2)	0.0549 (19)	0.0540 (17)	−0.0036 (16)	0.0154 (16)	−0.0014 (14)
C6	0.0578 (16)	0.0510 (16)	0.0478 (15)	0.0084 (13)	0.0272 (13)	0.0023 (13)
C7	0.0494 (15)	0.0502 (15)	0.0437 (14)	0.0035 (13)	0.0169 (12)	−0.0062 (12)
C8	0.0576 (17)	0.0519 (16)	0.0567 (16)	0.0043 (13)	0.0243 (13)	−0.0005 (13)
C9	0.075 (2)	0.0537 (18)	0.0669 (19)	0.0112 (16)	0.0198 (16)	0.0018 (14)
C10	0.068 (2)	0.070 (2)	0.077 (2)	0.0271 (17)	0.0189 (17)	−0.0024 (17)
C11	0.0471 (16)	0.086 (2)	0.073 (2)	0.0103 (16)	0.0255 (15)	−0.0120 (17)
C12	0.0529 (16)	0.0635 (18)	0.0618 (17)	−0.0021 (14)	0.0272 (14)	−0.0056 (14)
C13	0.0518 (15)	0.0479 (15)	0.0495 (15)	0.0058 (13)	0.0250 (12)	0.0010 (12)
C14	0.0594 (17)	0.0701 (18)	0.0408 (14)	0.0126 (15)	0.0190 (13)	0.0014 (13)
C15	0.0465 (15)	0.0670 (18)	0.0503 (15)	0.0106 (13)	0.0174 (12)	0.0012 (13)
C16	0.0466 (14)	0.0422 (13)	0.0429 (13)	−0.0006 (12)	0.0178 (11)	−0.0025 (11)
C17	0.0473 (14)	0.0470 (14)	0.0427 (13)	0.0035 (12)	0.0154 (11)	0.0027 (11)
C18	0.0459 (14)	0.0528 (15)	0.0522 (15)	0.0081 (12)	0.0227 (12)	0.0028 (12)
C19	0.0398 (13)	0.0449 (14)	0.0448 (14)	−0.0029 (11)	0.0151 (11)	−0.0030 (11)
C20	0.0495 (14)	0.0486 (15)	0.0407 (13)	−0.0009 (13)	0.0175 (11)	0.0047 (11)
C21	0.0432 (13)	0.0398 (14)	0.0459 (14)	−0.0055 (11)	0.0175 (11)	−0.0028 (11)
C22	0.0441 (13)	0.0425 (14)	0.0394 (13)	−0.0085 (11)	0.0168 (11)	−0.0066 (11)
C23	0.0470 (14)	0.0505 (15)	0.0427 (13)	−0.0033 (12)	0.0153 (11)	0.0069 (11)
C24	0.0446 (14)	0.0460 (15)	0.0513 (14)	0.0036 (12)	0.0181 (12)	0.0026 (12)
C25	0.0589 (16)	0.0408 (14)	0.0541 (15)	−0.0003 (13)	0.0232 (13)	−0.0004 (12)
C26	0.0572 (16)	0.0432 (15)	0.0604 (17)	0.0019 (13)	0.0246 (14)	−0.0075 (13)
C27	0.0579 (16)	0.0487 (16)	0.0585 (16)	−0.0062 (14)	0.0305 (13)	−0.0136 (13)

### Geometric parameters (Å, º)

**Table:**

O—N2	1.299 (2)	C12—H12A	0.9300
N1—C7	1.414 (3)	C13—C14	1.386 (3)
N1—C13	1.421 (3)	C13—C18	1.388 (3)
N1—C6	1.431 (3)	C14—C15	1.377 (3)
C1—C6	1.377 (4)	C14—H14A	0.9300
C1—C2	1.385 (4)	C15—C16	1.394 (3)
C1—H1A	0.9300	C15—H15A	0.9300
N2—C27	1.334 (3)	C16—C17	1.388 (3)
N2—C22	1.406 (3)	C16—C19	1.484 (3)
C2—C3	1.365 (4)	C17—C18	1.383 (3)
C2—H2A	0.9300	C17—H17A	0.9300
C3—C4	1.359 (4)	C18—H18A	0.9300
C3—H3A	0.9300	C19—C20	1.379 (3)
C4—C5	1.371 (4)	C19—C24	1.411 (3)
C4—H4A	0.9300	C20—C21	1.417 (3)
C5—C6	1.371 (4)	C20—H20A	0.9300
C5—H5A	0.9300	C21—C22	1.399 (3)
C7—C8	1.383 (3)	C21—C25	1.414 (3)
C7—C12	1.400 (3)	C22—C23	1.402 (3)
C8—C9	1.380 (4)	C23—C24	1.359 (3)
C8—H8A	0.9300	C23—H23A	0.9300
C9—C10	1.369 (4)	C24—H24A	0.9300
C9—H9A	0.9300	C25—C26	1.355 (3)
C10—C11	1.363 (4)	C25—H25A	0.9300
C10—H10A	0.9300	C26—C27	1.386 (4)
C11—C12	1.384 (4)	C26—H26A	0.9300
C11—H11A	0.9300	C27—H27A	0.9300
			
C7—N1—C13	122.3 (2)	C18—C13—N1	122.2 (2)
C7—N1—C6	120.13 (19)	C15—C14—C13	121.1 (2)
C13—N1—C6	117.5 (2)	C15—C14—H14A	119.5
C6—C1—C2	119.9 (3)	C13—C14—H14A	119.5
C6—C1—H1A	120.0	C14—C15—C16	121.3 (2)
C2—C1—H1A	120.0	C14—C15—H15A	119.3
O—N2—C27	121.6 (2)	C16—C15—H15A	119.3
O—N2—C22	119.6 (2)	C17—C16—C15	117.3 (2)
C27—N2—C22	118.9 (2)	C17—C16—C19	121.4 (2)
C3—C2—C1	120.7 (3)	C15—C16—C19	121.4 (2)
C3—C2—H2A	119.7	C18—C17—C16	121.6 (2)
C1—C2—H2A	119.7	C18—C17—H17A	119.2
C4—C3—C2	119.2 (3)	C16—C17—H17A	119.2
C4—C3—H3A	120.4	C17—C18—C13	120.6 (2)
C2—C3—H3A	120.4	C17—C18—H18A	119.7
C3—C4—C5	120.7 (3)	C13—C18—H18A	119.7
C3—C4—H4A	119.6	C20—C19—C24	117.9 (2)
C5—C4—H4A	119.6	C20—C19—C16	121.2 (2)
C4—C5—C6	120.8 (3)	C24—C19—C16	120.9 (2)
C4—C5—H5A	119.6	C19—C20—C21	121.8 (2)
C6—C5—H5A	119.6	C19—C20—H20A	119.1
C5—C6—C1	118.7 (3)	C21—C20—H20A	119.1
C5—C6—N1	120.0 (2)	C22—C21—C25	119.7 (2)
C1—C6—N1	121.3 (3)	C22—C21—C20	117.8 (2)
C8—C7—C12	118.3 (2)	C25—C21—C20	122.5 (2)
C8—C7—N1	121.5 (2)	C21—C22—C23	121.0 (2)
C12—C7—N1	120.2 (2)	C21—C22—N2	119.7 (2)
C9—C8—C7	120.9 (2)	C23—C22—N2	119.3 (2)
C9—C8—H8A	119.5	C24—C23—C22	119.2 (2)
C7—C8—H8A	119.5	C24—C23—H23A	120.4
C10—C9—C8	120.1 (3)	C22—C23—H23A	120.4
C10—C9—H9A	119.9	C23—C24—C19	122.3 (2)
C8—C9—H9A	119.9	C23—C24—H24A	118.9
C11—C10—C9	120.1 (3)	C19—C24—H24A	118.9
C11—C10—H10A	120.0	C26—C25—C21	118.5 (2)
C9—C10—H10A	120.0	C26—C25—H25A	120.8
C10—C11—C12	120.7 (3)	C21—C25—H25A	120.8
C10—C11—H11A	119.7	C25—C26—C27	121.0 (2)
C12—C11—H11A	119.7	C25—C26—H26A	119.5
C11—C12—C7	119.9 (3)	C27—C26—H26A	119.5
C11—C12—H12A	120.0	N2—C27—C26	122.2 (2)
C7—C12—H12A	120.0	N2—C27—H27A	118.9
C14—C13—C18	118.1 (2)	C26—C27—H27A	118.9
C14—C13—N1	119.6 (2)		
			
C6—C1—C2—C3	−0.1 (4)	C15—C16—C17—C18	−1.3 (4)
C1—C2—C3—C4	0.3 (5)	C19—C16—C17—C18	177.5 (2)
C2—C3—C4—C5	−0.1 (5)	C16—C17—C18—C13	0.9 (4)
C3—C4—C5—C6	−0.4 (5)	C14—C13—C18—C17	−0.4 (4)
C4—C5—C6—C1	0.5 (4)	N1—C13—C18—C17	−178.2 (2)
C4—C5—C6—N1	179.3 (3)	C17—C16—C19—C20	−144.3 (2)
C2—C1—C6—C5	−0.3 (4)	C15—C16—C19—C20	34.5 (4)
C2—C1—C6—N1	−179.1 (2)	C17—C16—C19—C24	35.0 (3)
C7—N1—C6—C5	117.4 (3)	C15—C16—C19—C24	−146.2 (2)
C13—N1—C6—C5	−63.9 (3)	C24—C19—C20—C21	1.2 (3)
C7—N1—C6—C1	−63.8 (3)	C16—C19—C20—C21	−179.4 (2)
C13—N1—C6—C1	114.9 (3)	C19—C20—C21—C22	−1.7 (3)
C13—N1—C7—C8	−23.1 (4)	C19—C20—C21—C25	177.6 (2)
C6—N1—C7—C8	155.5 (2)	C25—C21—C22—C23	−178.1 (2)
C13—N1—C7—C12	158.5 (2)	C20—C21—C22—C23	1.2 (3)
C6—N1—C7—C12	−22.9 (4)	C25—C21—C22—N2	1.1 (3)
C12—C7—C8—C9	−0.6 (4)	C20—C21—C22—N2	−179.6 (2)
N1—C7—C8—C9	−179.0 (2)	O—N2—C22—C21	178.3 (2)
C7—C8—C9—C10	1.4 (4)	C27—N2—C22—C21	−1.7 (3)
C8—C9—C10—C11	−1.2 (5)	O—N2—C22—C23	−2.5 (3)
C9—C10—C11—C12	0.3 (5)	C27—N2—C22—C23	177.5 (2)
C10—C11—C12—C7	0.5 (4)	C21—C22—C23—C24	−0.3 (4)
C8—C7—C12—C11	−0.4 (4)	N2—C22—C23—C24	−179.5 (2)
N1—C7—C12—C11	178.0 (2)	C22—C23—C24—C19	−0.3 (4)
C7—N1—C13—C14	141.4 (3)	C20—C19—C24—C23	−0.2 (3)
C6—N1—C13—C14	−37.3 (4)	C16—C19—C24—C23	−179.6 (2)
C7—N1—C13—C18	−40.8 (4)	C22—C21—C25—C26	0.0 (3)
C6—N1—C13—C18	140.5 (3)	C20—C21—C25—C26	−179.3 (2)
C18—C13—C14—C15	0.4 (4)	C21—C25—C26—C27	−0.4 (4)
N1—C13—C14—C15	178.3 (2)	O—N2—C27—C26	−178.7 (2)
C13—C14—C15—C16	−0.8 (4)	C22—N2—C27—C26	1.3 (4)
C14—C15—C16—C17	1.3 (4)	C25—C26—C27—N2	−0.2 (4)
C14—C15—C16—C19	−177.6 (2)		

### Hydrogen-bond geometry (Å, º)

Cg3 is the centroid of the C7–C12 benzene ring.

**Table:**

*D*—H···*A*	*D*—H	H···*A*	*D*···*A*	*D*—H···*A*
C17—H17*A*···*Cg*3^i^	0.93	2.76	3.640 (3)	158

Symmetry code: (i) *x*, −*y*−1/2, *z*−3/2.
